# Emodin Combined with Multiple-Low-Frequency, Low-Intensity Ultrasound To Relieve Osteomyelitis through Sonoantimicrobial Chemotherapy

**DOI:** 10.1128/spectrum.00544-22

**Published:** 2022-09-07

**Authors:** Feng Lu, Xinhui Wu, Huiqun Hu, Zixuan He, Jiacheng Sun, Jiapeng Zhang, Xiaoting Song, Xiangang Jin, Guofu Chen

**Affiliations:** a Department of Orthopedics, Taizhou Hospital of Zhejiang Province, Zhejiang University, Linhai, China; b Zhejiang University School of Medicine, Hangzhou, China; c Wenzhou Medical University, Wenzhou, China; d Department of Orthopedics, Taizhou Hospital Affiliated with Wenzhou Medical University, Linhai, China; e Department of Infectious Diseases, The Second Affiliated Hospital, Zhejiang University School of Medicine, Hangzhou, China; Riverside University Health System, Medical Center, University of California

**Keywords:** osteomyelitis, emodin, low-frequency ultrasound, low-intensity ultrasound, biofilm, sonoantimicrobial chemotherapy

## Abstract

Treatment of osteomyelitis is still challenging, as conventional antibiotic therapy is limited by the emergence of resistant strains and the formation of biofilms. Sonoantimicrobial chemotherapy (SACT) is a novel therapy of low-frequency and low-intensity ultrasound (LFLIU) combined with a sonosensitizer. Therefore, in our study, a sonosensitizer named emodin (EM) was proposed to be combined with LFLIU to relieve acute osteomyelitis caused by methicillin-resistant Staphylococcus aureus (MRSA) through antibacterial and antibiofilm effects. The efficiencies of different intensities of ultrasound, including single (S-LFLIU, 15 min) and multiple ultrasound (M-LFLIU, 3 times for 5 min at 4-h intervals), against bacteria and biofilms were compared, contributing to developing the best treatment regimen. Our results demonstrated that EM plus S-LFLIU or M-LFLIU (EM+S-LFLIU or EM+M-LFLIU) had significant combined bactericidal and antibiofilm effects, with EM+M-LFLIU in particular exhibiting superior antibiofilm performance. Furthermore, it was suggested that EM+M-LFLIU could produce a large amount of reactive oxygen species (ROS), destroy the integrity of the bacterial membrane and cell wall, and downregulate the expression of genes involved in oxidative stress, membrane wall synthesis, and bacterial virulence, as well as that of other related genes (*agrB*, *pbp3*, *sgtB*, *gmk*, *zwf*, and *msrA*). *In vivo* studies, micro-computed tomography (micro-CT), hematoxylin and eosin (H&E) staining, enzyme-linked immunosorbent assay (ELISA), and bacterial quantification of bone tissue indicated that EM+M-LFLIU could also relieve osteomyelitis due to MRSA infection. Our work proffers an original approach to bacterial osteomyelitis treatment that weakens drug-resistant bacteria and suppresses and degrades biofilm formation through SACT, which may provide new prospects for clinical treatment.

**IMPORTANCE** Antibiotic therapy is the first choice for clinical treatment of osteomyelitis, but the formation of bacterial biofilms and the emergence of many drug-resistant strains also create an urgent need to find an alternative treatment to effectively eliminate the infection. Recently, LFLIU has come to be considered a safe and promising method of debridement and antibacterial therapy. In this study, we found that ultrasound and EM have a significant combined antibacterial effect *in vivo* and *in vitro*, which may play an antibacterial role by stimulating the production of ROS, destroying the bacterial cell wall, and inhibiting the expression of related genes. Our study expands the body of knowledge on the antibacterial effect of drugs—specifically emodin (EM)—through combined physiotherapy. If successfully integrated into clinical practice, these methods may reduce the burden of high concentrations of drugs needed to treat bacterial biofilms and avoid the growing resistance of bacteria to antibiotics.

## INTRODUCTION

Limb injuries caused by various high-energy external forces are mostly open injuries and often involve bone. If the wound is deep and the bone is damaged, the subcutaneous soft tissue and bone will be exposed to the external environment, allowing bacteria to enter the bone and potentially cause acute osteomyelitis, a deep tissue infection that usually causes abscesses, organ infections, and sepsis. In severe cases, it can be life-threatening (1). It has been reported that up to 30% of high-risk open fractures will be infected (2, 3). Furthermore, invasive bacteria will gradually form localized biofilms, which are organized bacterial populations encapsulated by extracellular polymeric substances (EPS) (4). Antibiotic treatment is by far the most common treatment method, but the dosages of antibiotics required to eliminate bacteria in a biofilm are 500 to 5,000 times more than those required for free-floating or planktonic bacteria, which is beyond the human body’s ability to bear (5). In addition, antibiotic treatment often fails to completely eradicate the biofilm, leading to recurrent biofilm-associated infections (6, 7). The emergence of many drug-resistant strains also makes it imperative to find an alternative therapy to effectively remove biofilms and clear the infections despite drug resistance in the population (8).

Physical methods are important auxiliary methods in the struggle against bacterial drug resistance. These physical methods include the use of magnetic fields, electric fields, and UV light. Among them, low-frequency (20 kHz to 1 MHz) and low-intensity (20 to 1,000 mW/cm^2^) ultrasound (LFLIU) is a safe and promising debridement and antibacterial treatment (9, 10). The bactericidal effect of LFLIU alone on bacteria is low, but several studies have shown that the combined application of LFLIU and antibiotics can produce an excellent bactericidal effect; that is, LFLIU can enhance the bactericidal ability of antibiotics, whether on planktonic bacteria or bacterial biofilms (11–13). The stable cavitation of LFLIU generates numerous channels in the bacterial membrane and the extracellular matrix of biofilm that promote the rapid entry of antibiotics into the bacteria, and the promoted entry of antimicrobials into bacterial cells or biofilm results in increasing the efficacy of antibiotics. Meanwhile, with the entry of more oxygen and nutrients, the sensitivity of bacteria to antibiotics can be gradually restored (14, 15). However, the use of antibiotics retains the risk of increasing the incidence of microbial drug resistance. Therefore, developing a novel antibiotic alternative in combating the emergence of drug-resistant strains and biofilms is still a great clinical challenge.

Sonoantimicrobial chemotherapy (SACT) is an emerging antimicrobial method based on a combination of sonosensitizers and LFLIU to enhance the inactivation of the target cells. The possible mechanisms of SACT include generating cytotoxic reactive species, such as reactive oxygen species (ROS), triggering lipid oxidation, and breaking down bacterial cell membrane structures (16, 17). In addition, LFLIU can act on microorganisms in deep tissues, preferentially focusing on a specific local area to activate the sonosensitizer and ultimately exert local toxicity (18). Meanwhile, emodin (EM) is a natural anthraquinone derivative used as an alternative antimicrobial treatment. Furthermore, as a sonosensitizer, EM also has the potential to have combined effects with LFLIU, reportedly including anti-inflammatory, antibacterial, and anticancer effects (19–21).

Here, we explored the possible combined effects of LFLIU and EM on planktonic bacteria and biofilm colonies. The possible influencing factors, including ultrasonic frequency (single-frequency LFLIU [S-LFLIU] and multifrequency LFLIU [M-LFLIU]), ultrasonic intensity, EM concentration, and strain, were analyzed *in vitro*. The therapeutic effects of the regimens were then observed in a methicillin-resistant Staphylococcus aureus (MRSA) bone infection *in vivo*. The results showed that EM and LFLIU have excellent combined effects, whether on planktonic bacteria or biofilms. LFLIU greatly improved the antibacterial and antibiofilm effects of EM. Furthermore, the combined application of LFLIU and EM was observed to effectively treat acute osteomyelitis, indicating its effective antibacterial ability *in vivo*.

## RESULTS

### Antibacterial efficiencies of different intensities of LFLIU and different concentrations of EM against planktonic bacterial cells of MSSA and MRSA.

**FIG 1:**
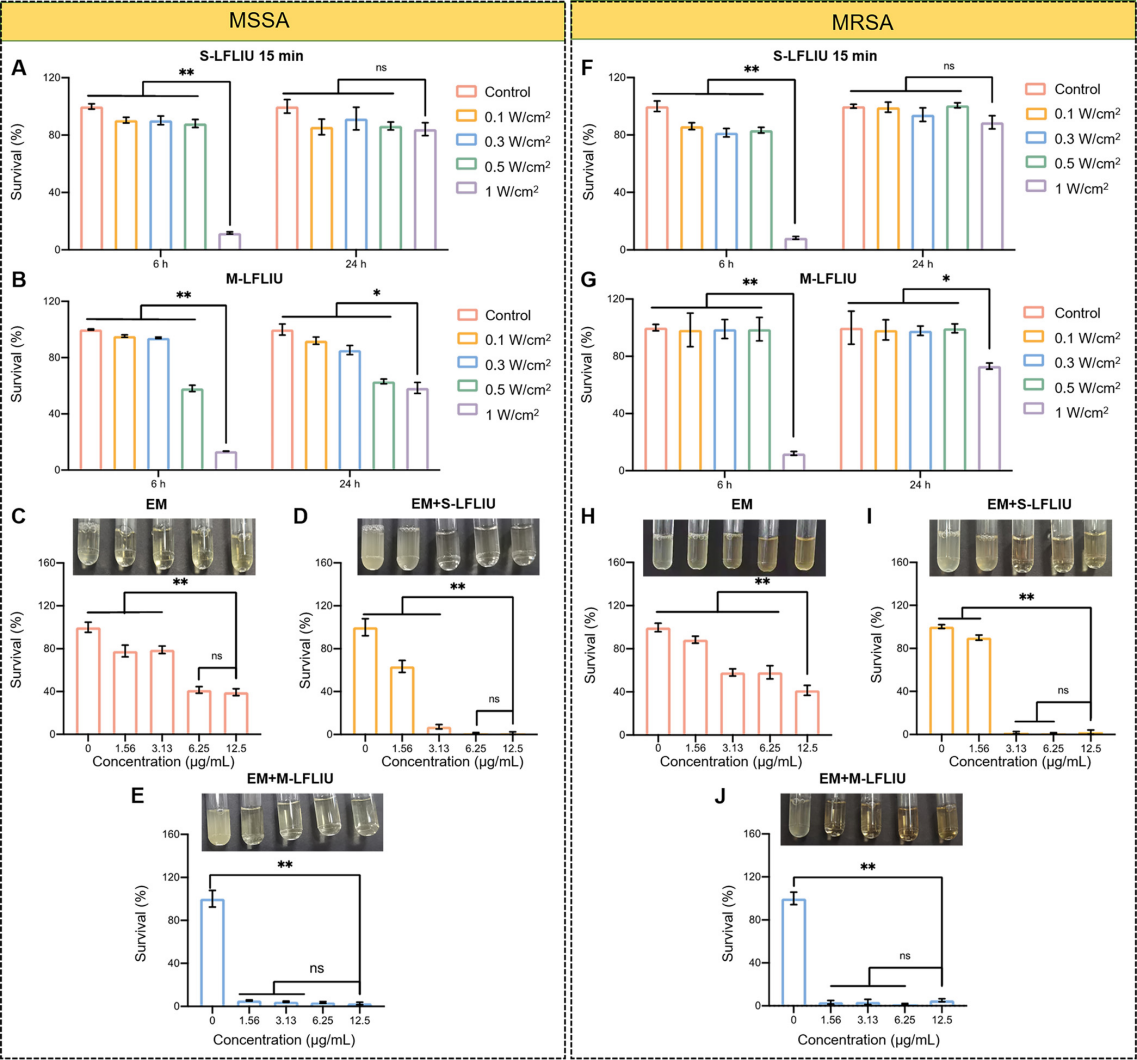
Survival rates of MSSA and MRSA bacterial cells after EM and/or LFLIU treatment. (A to E) Survival rates of MSSA treated with S-LFLIU for 15 min (A) or M-LFLIU 3 times for 5 min at 4-h intervals (B) at different frequencies after 6 h and 24 h or with different concentrations of EM (C), EM+S-LFLIU (D), or EM+M-LFLIU (E) at 37°C after 24 h. (F to J) Percentages of survival of MRSA cells after treatment with S-LFLIU for 15 min (F) or M-LFLIU 3 times for 5 min at 4-h intervals (G) at different frequencies after 6 h and 24 h or with different concentrations of EM (H), EM+S-LFLIU (I), or EM+M-LFLIU (J) at 37°C after 24 h.

Methicillin-sensitive S. aureus (MSSA) was treated with different intensities of S-LFLIU for 15 min. The survival rates of MSSA were evaluated using the value for optical density at 600 nm (OD_600_) after 6 h and 24 h of incubation, respectively. When the intensity was raised to 1 W/cm^2^, the survival rate decreased significantly after 6 h, but there was no discernible difference after 24 h (Fig. 1A). M-LFLIU (5 min every 4 h) therapy yielded similar results (Fig. 1B), indicating that the antibacterial effect of ultrasound treatment was temporary. Similar results were seen in parallel experiments on MRSA (Fig. 1F and G). Based on these results, ultrasonic irradiation at 1 W/cm^2^ may provide favorable conditions for bacterial growth inhibition, and thus, this intensity was used in the subsequent experiments.

### Antibacterial activities of EM combined with S-LFLIU or M-LFLIU against planktonic bacterial cells of MSSA and MRSA.

Using MSSA planktonic cells in Luria-Bertani (LB) broth, we investigated the antibacterial activity of EM alone, as well as EM in combination with S-LFLIU (1 W/cm^2^) or M-LFLIU (1 W/cm^2^). The survival rate of MSSA decreased as the concentration of EM was increased. Even when the concentration was elevated to 12.5 μg/mL, the survival rate was still as high as 39.4%. (Fig. 1C). The bacterial survival rate was clearly reduced in EM coupled with S-LFLIU or M-LFLIU, where it was less than 10%, with the bacterial solution becoming clear after 24 h of incubation (Fig. 1D and E). Simultaneous MRSA trials yielded similar results (Fig. 1F to J). These findings imply that LFLIU can significantly improve EM’s bactericidal ability.

**FIG 2:**
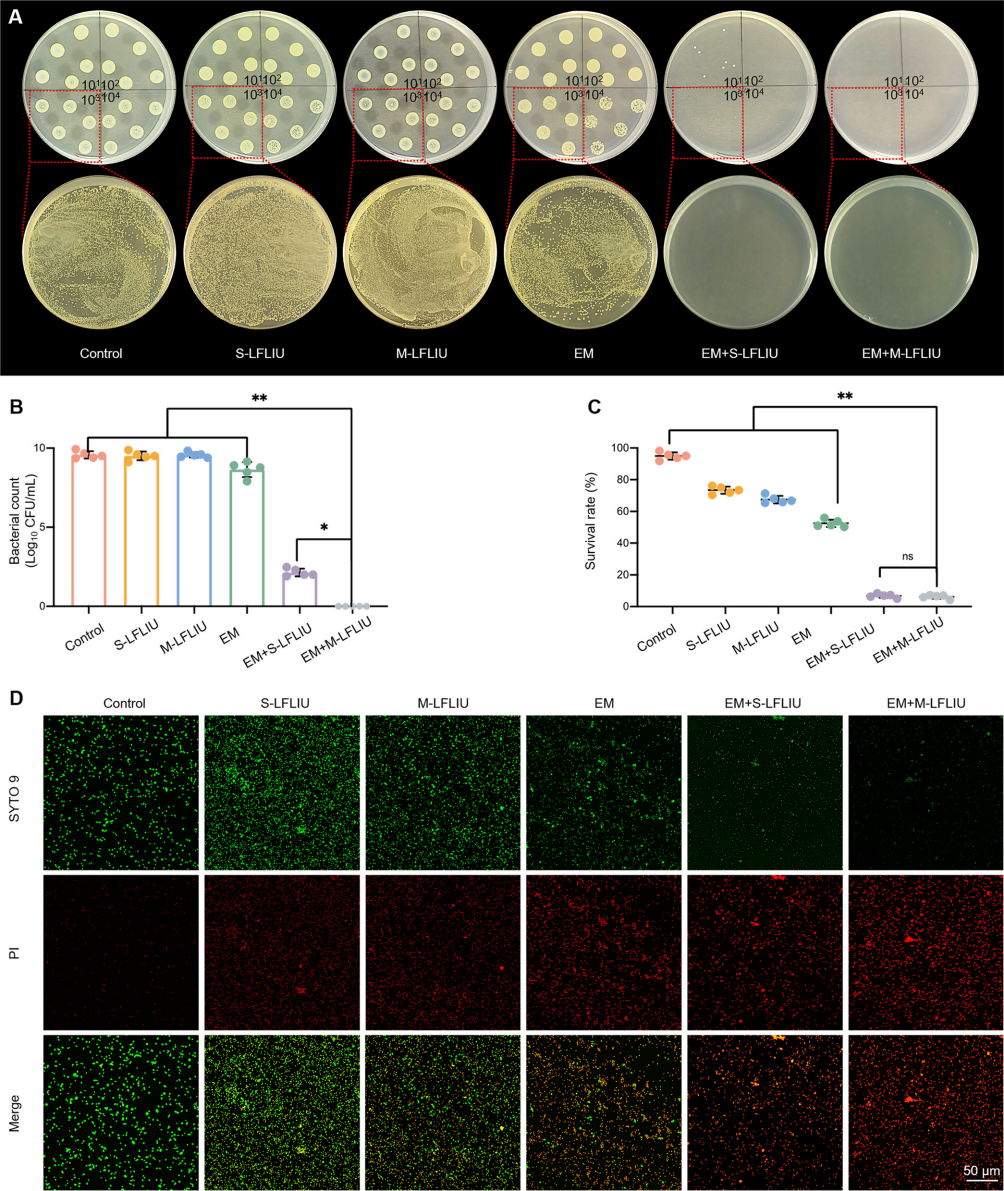
Antibacterial activity of S-LFLIU, M-LFLIU, EM, EM+S-LFLIU, or EM+M-LFLIU. (A and B) MRSA cells treated with S-LFLIU, M-LFLIU, EM, EM+S-LFLIU, or EM+M-LFLIU at 37°C for 24 h (S-LFLIU, 15 min; M-LFLIU, 5 min every 4 h TID) were grown on LB agar plates (top, diluted 10^1^, 10^2^, 10^3^, and 10^4^ times; bottom, diluted 10^3^ times) (A), and the numbers of colonies were quantified (B). (C and D) After the same treatment, MRSA cells were stained with live/dead dye and photographed under a confocal microscope (C), and the survival rates were calculated according to the red and green fluorescence intensities (D).

To further study the antibacterial activity of EM plus S-LFLIU or M-LFLIU, we evaluated the viable bacterial counts of the following treatment groups on LB agar plates: control, S-LFLIU, M-LIFLIU, EM, EM plus S-LFLIU (EM+S-LFLIU), and EM+M-LFLIU (Fig. 2A and B). The control, S-LFLIU, and M-LIFLIU groups did not differ in any way. The number of viable bacteria was reduced in the EM group, and both the EM+S-LFLIU and EM+M-LFLIU groups had relatively few surviving colonies. There was no significant difference between S-LFLIU and M-LFLIU in terms of enhancing the bactericidal impact of EM, indicating a considerable combined effect of EM and LFLIU.

The proportion of live bacteria to the total bacterial population was not different in the control group, the S-LFLIU group, or the M-LIFLIU group, but it was lower in the EM group. The proportion of live bacteria was found to be lower in the EM+S-LFLIU and EM+M-LFLIU groups. The survival rates of the bacteria were then determined, and both the EM+S-LFLIU and EM+M-LFLIU groups had significantly diminished survival rates (Fig. 2C and D). This demonstrates that EM and LFLIU have a combined bactericidal effect. Furthermore, we compared the antibacterial efficiencies of ampicillin (AMP) and EM+M-LFLIU against MRSA. As shown by the results in Fig. S1 in the supplemental material, the MIC of AMP was <62.5 μg/mL, much higher than the MIC of EM+M-LFLIU, indicating that the antibacterial effect of EM+ M-LFLIU is greater than that of AMP.

### Combined activity of S-LFLIU or M-LFLIU plus EM against MRSA biofilm.

**FIG 3:**
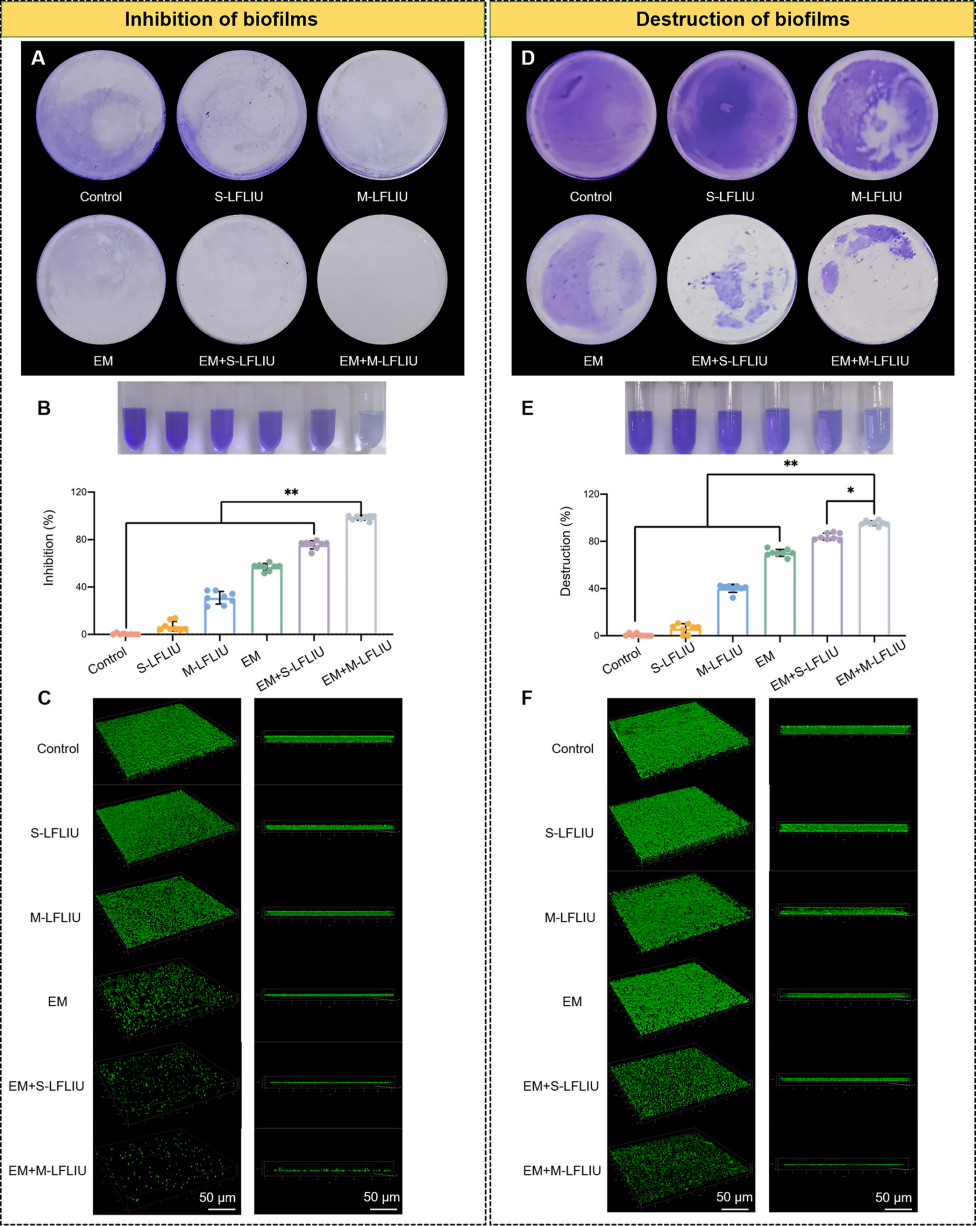
Antibiofilm activity of S-LFLIU, M-LFLIU, EM, EM+S-LFLIU, or EM+M-LFLIU. (A and B) MRSA cells were treated with S-LFLIU, M-LFLIU, EM, EM+S-LFLIU, or EM+M-LFLIU at 37°C for 24 h (S-LFLIU, 15 min; M-LFLIU, 3 times for 5 min at 4-h intervals), and then the bacterial suspension was discarded, residual biofilms were stained by crystal violet staining (A), and the rates of inhibition of MRSA biofilm formation were calculated (bottom) (top, crystal violet-stained biofilms dissolved in ethanol) (B). (C) Cover glasses were placed on the bottom of 24-well plates, and after the same treatments, biofilms were stained with SYTO 9 and 3-D fluorescence images of MRSA biofilms were taken by using a laser confocal microscope (left, top view; right, side view). (D and E) MRSA was incubated at 37°C for 24 h to form mature biofilms, and the mature biofilms were treated with blank LB medium, S-LFLIU, M-LFLIU, EM, EM+S-LFLIU, or EM+M-LFLIU at 37°C for 24 h (S-LFLIU, 15 min; M-LFLIU, 3 times for 5 min at 4-h intervals) and then stained with crystal violet (D), and the rates of destruction of MRSA biofilm formation were calculated (bottom) (top, photo of crystal violet stained biofilm dissolved in ethanol) (E). (F) Biofilms were formed on the glasses, treated with the same treatments, and stained with SYTO 9 to obtain 3-D fluorescence images of mature MRSA biofilms (left, top view; right, side view).

Considering the significant bactericidal activity of EM plus LFLIU against planktonic MRSA, we next studied its effect on MRSA biofilms. We treated bacterial biofilms at two stages, early (0 h) and late (24 h), to see what impact the treatment had on biofilm development and biofilm destruction, respectively. After treatment with S-LFLIU, M-LFLIU, EM, EM+S-LFLIU, or EM+M-LFLIU and incubation for 24 h, the formed biofilms were stained using 1% crystal violet and SYTO 9 dye. As seen by the results in Fig. 3A and B, the stained biofilms in the S-LFLIU group were not noticeably thinner. The biofilms in the M-LFLIU and EM groups were marginally thinner than those in the EM+S-LFLIU and EM+M-LFLIU groups, while the biofilms in the EM+S-LFLIU and EM+M-LFLIU groups were significantly thinner. The inhibition rates for the four groups were 7.0%, 30.9%, 56.8%, 75.6%, and 98.5%, respectively. Interestingly, the produced biofilms might be degraded to various degrees after the same treatment, with destruction rates of 6.1%, 40.2%, 69.9%, 83.6%, and 95.5% for the same groups, respectively (Fig. 3D and E).

Changes in the three-dimensional (3-D) structure of biofilms were detected using confocal microscopy after various treatments. The 3-D structures of biofilms following early- and late-stage treatments, respectively, are depicted in Fig. 3C and F. The EM+M-LFLIU group exhibited the least green signal and thinnest thickness, implying that EM+M-LFLIU had the best antibiofilm effect.

**FIG 4:**
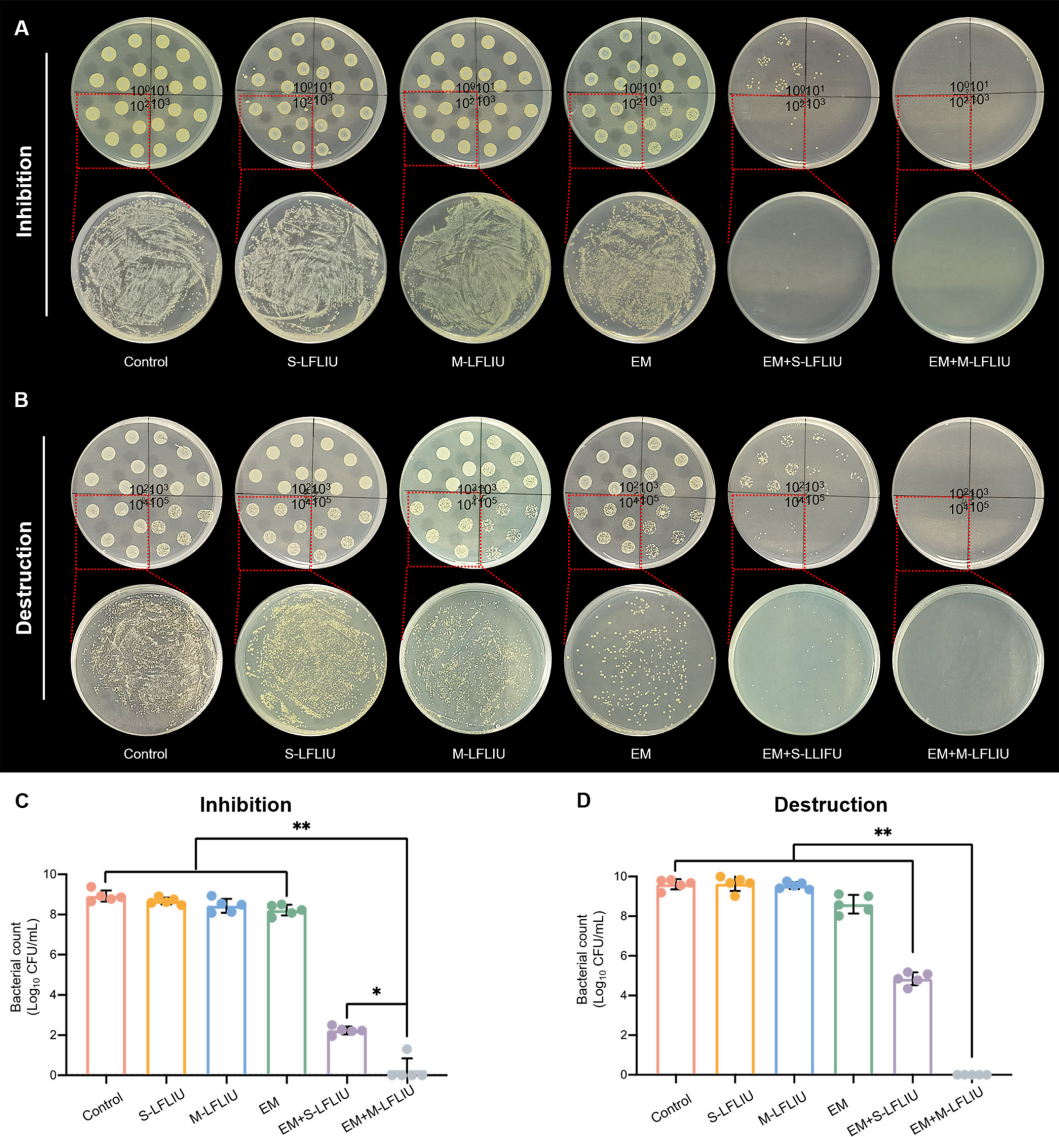
Living bacterial cell activity in biofilms after treatment with S-LFLIU, M-LFLIU, EM, EM+S-LFLIU, or EM+M-LFLIU. (A) MRSA cells were treated with S-LFLIU, M-LFLIU, EM, EM+S-LFLIU, or EM+M-LFLIU at 37°C for 24 h (S-LFLIU, 15 min; M-LFLIU, 3 times for 5 min at 4-h intervals), and residual biofilms were collected, dispersed, and grown on LB agar plates (top, diluted 10^0^, 10^1^, 10^2^, and 10^3^ times; bottom, diluted 10^2^ times). (B) MRSA cells were incubated at 37°C for 24 h to form mature biofilms, and the mature biofilms were treated with blank LB medium, S-LFLIU, M-LFLIU, EM, EM+S-LFLIU, or EM+M-LFLIU at 37°C for 24 h (S-LFLIU, 15 min; M-LFLIU, 3 times for 5 min at 4-h intervals) and the living MRSA cells grown on LB agar plates (top, diluted 10^2^, 10^3^, 10^4^, and 10^5^ times; bottom, diluted 10^4^ times). (C and D) The numbers of living bacteria in both immature (C) and mature (D) biofilms were quantified.

To assess the bacterial viability in treated MRSA biofilms, bacterial plate counting was used. Photographs of colonies growing on plates are shown in Fig. 4A and B, whereas Fig. 4C and D provide quantitative colony counts. The numbers of colonies in the S-LFLIU, M-LFLIU, and EM groups were clearly reduced compared to the number in the blank LB medium treatment, with even higher reductions in the EM+S-LFLIU and EM+M-LFLIU groups, regardless of whether the biofilm was treated at the early or late stage. Furthermore, after treatment with EM+M-LFLIU, almost no bacteria were identified, indicating that EM plus LFLIU had outstanding antibiofilm properties. These findings suggest that EM and LFLIU have combined antibiofilm action against MRSA, with EM+M-LFLIU outperforming EM+S-LFLIU in this regard. As a result, it was hypothesized that EM+M-LFLIU could inhibit biofilm development and remove established biofilms more effectively than EM+S-LFLIU. In addition, we further compared the antibiofilm efficiencies of AMP and EM+M-LFLIU against MRSA. The experimental results of 3-D structure imaging of biofilm, crystal violet staining of biofilm, and quantitative analysis of viable bacteria in biofilm all showed that the effect of EM+M-LFLIU on inhibiting biofilm formation and destroying mature biofilm was stronger than that of AMP (Fig. S2 to S4). It is known that the threat of drug resistance is becoming more and more serious, so we decided to determine the resistance of MRSA to EM+M-LFLIU and to AMP. As shown by the results in Fig. S5, after 6 generations of culture, the MIC of MRSA incubated with AMP increased to 128,000 μg/mL, 2,048 times that of the first generation. However, the MIC of 6th-generation MRSA treated with EM+M-LFLIU only increased to 12.5 μg/mL, 8 times that of the first generation. These results suggested that EM+M-LFLIU may be expected to improve the problem of drug resistance.

### Potential antibacterial mechanism of EM plus LFLIU.

Our earlier experiments confirmed the excellent antibacterial and antibiofilm effects of EM plus LFLIU. To examine its potential antibacterial mechanism, evaluations of ROS production, transmission electron microscopy (TEM) imaging, and specific gene expression were performed.

**FIG 5:**
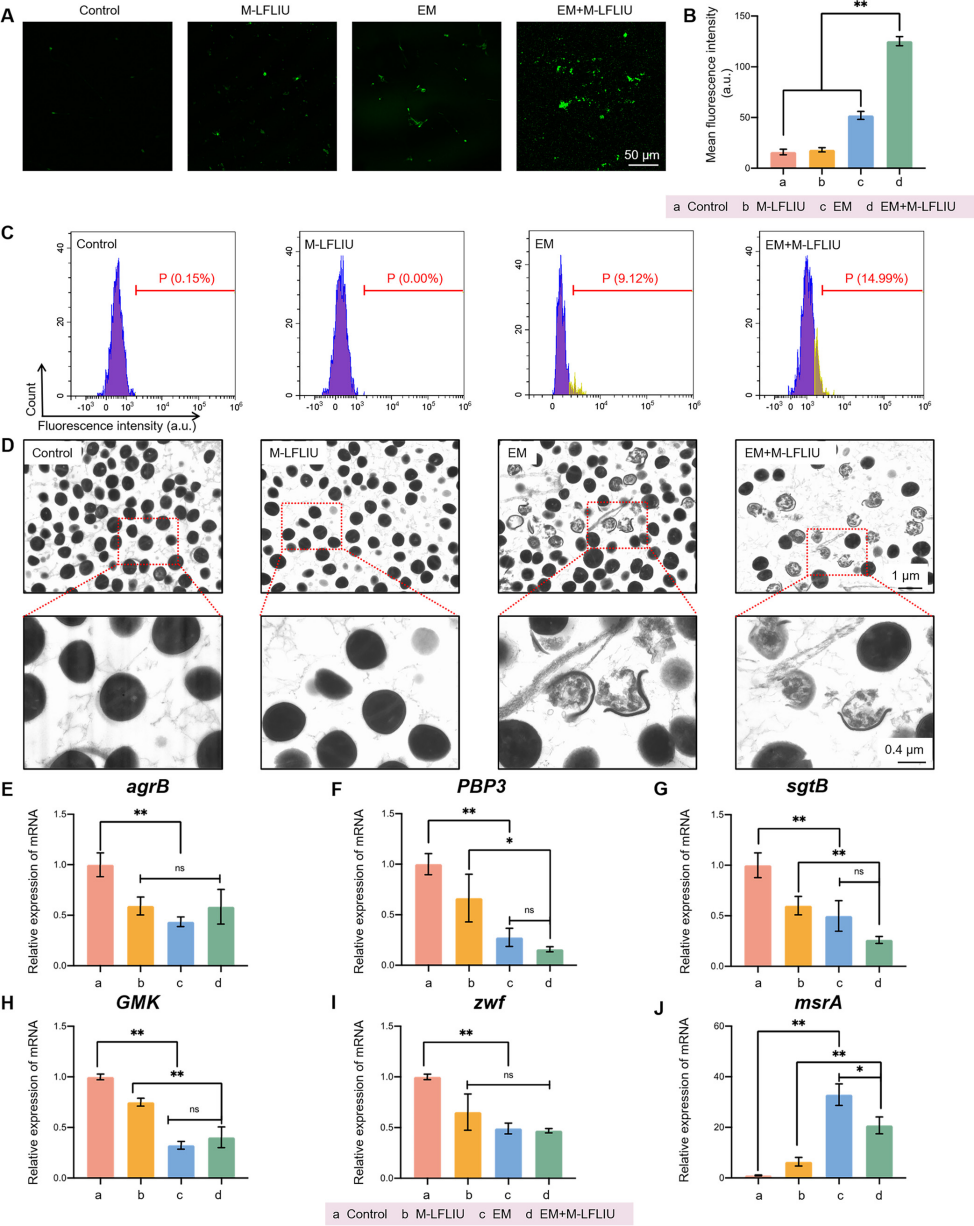
Potential antibacterial mechanism of EM plus LFLIU. (A and B) MRSA cells were treated with M-LFLIU, EM, or EM+M-LFLIU at 37°C for 24 h (M-LFLIU, 3 times for 5 min at 4-h intervals), stained with ROS probe, and photographed under a laser confocal microscope (A), and the fluorescence intensities were quantified (a.u., arbitrary units) (B). (C) After the same treatments, the rates of positivity of ROS production in the different groups were detected by flow cytometer. (D) MRSA cells in the different groups were treated for 12 h and the photographed under a TEM. Boxed areas in the top views are enlarged below. (E to J) After treatment with EM, M-LFLIU, or EM+M-LFLIU (EM, 6.25 μg/mL; M-LFLIU, 1 W/cm^2^), mRNA was extracted from MRSA cells and reverse transcribed into cDNA, and the expression levels of *agrB* (E), *pbp3* (F), *sgtB* (G), *gmk* (H), *zwf* (I), and *msrA* (J) were measured.

The results shown in Fig. 5A and B show that when bacteria were treated with EM+M-LFLIU, the greatest ROS signal was seen, followed by a weak ROS signal in EM alone and essentially none in the control and M-LFLIU groups. Similarly, the flow cytometry data (Fig. 5C) indicate that a substantial quantity of ROS was created in the EM+M-LFLIU group, indicating that it was involved in the sterilizing process.

To learn what the morphological changes of the MRSA bacterial cell were in different treatment groups, TEM was conducted (Fig. 5D). The bacteria in the control and M-LFLIU groups were found to be unaffected, and there was no discernible difference between the two groups. However, the bacteria treated with EM and EM plus M-LFLIU lost bacterial wall integrity, and their cell contents leaked to a large extent. Additionally, the EM plus M-LFLIU group had fewer bacteria and a larger proportion of bacteria presenting with bacterial membrane rupture, indicating that EM in combination with LFLIU could damage the integrity of the cell membrane and wall.

Furthermore, certain genes involved in bacterial growth, bacterial virulence, and oxidative stress were studied and their expression was verified by quantitative PCR. For example, *agrB*, a member of the *agr* family, is responsible for encoding accessory gene regulator protein B, located in the plasma membrane, which is associated with vancomycin sensitivity and with the virulence of MSSA (22–25). In regulating bacterial growth and synthesis, penicillin-binding protein 3 (PBP3) is involved in the final stage of peptidoglycan biosynthesis in MSSA, thus playing a role in the formation of the cell wall. Meanwhile, monofunctional glycosyltransferase (MGT) has been shown to play a key role in the extension of the peptidoglycan chain. *pbp3* and *sgtB*, respectively, are the genes responsible for encoding the above-described two proteins (23, 26, 27). *gmk*, which encodes the cytoplasmic guanosine kinase, is involved in the synthesis of nucleotide precursors and the indirect regulation of DNA and RNA synthesis. Inhibition of *gmk* can affect bacterial growth; therefore, it is a potential target for new antimicrobial agents against MSSA (23, 28). In terms of bacterial regulation of oxidative stress, we found that *zwf* and *msrA* play an important role in protecting cells from oxidative stress (23). The upregulation of *zwf* has been proven to be a response to oxidative stress, heat stress, and even virulence (29–31). On the other hand, *msrA* encodes methionine *S*-sulfoxide reductase, the downregulation of which may affect the resistance to oxidative stress (32, 33). Then, in terms of bacterial virulence- and oxidative stress-related genes, our data demonstrated that M-LFLIU and EM might decrease their expression to a certain level, but there was no apparent combined impact. EM suppresses the expression of cell wall synthesis-related genes, and ultrasound appears to have a combinatory effect (Fig. 5E to J), a finding that was also mutually verified by the previous TEM results.

In summary, LFLIU combined with EM appears to have antibacterial effects on MRSA by producing ROS and damaging the bacterial cell wall while also inhibiting the expression of genes involved in bacterial cell wall synthesis. Additionally, EM+M-LFLIU may also diminish MRSA virulence, reduce its toxic and adverse effects on the host, weaken its resistance to oxidative stress, and improve the bactericidal effect of ROS.

### *In vivo* effect of EM+M-LFLIU on osteomyelitis caused by MRSA.

**FIG 6:**
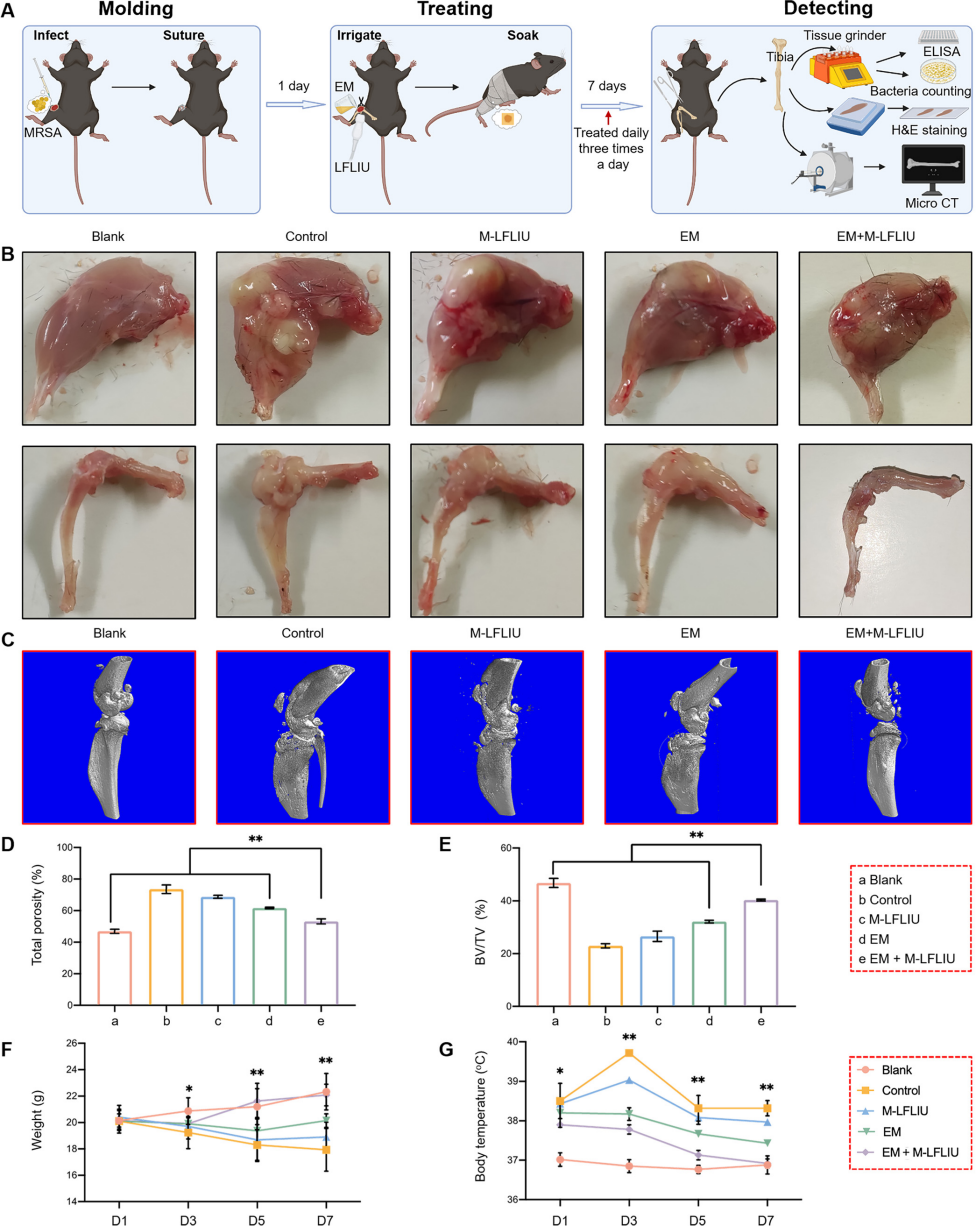
Effects of different treatments on bone protection and general condition of mice with acute osteomyelitis. (A) The right knee joints of the mice were opened, holes punched in the tibial plateau, MRSA bacterial cell solution injected, and finally, the wounds were sutured. The treatment began 1 day later: the wounds were washed with EM solution for 5 min, and the ultrasound group was treated with ultrasound at the same time, followed by application of a wet EM compress and simple bandaging, three times a day. (B) After 7 days, the leg tissues of mice were observed and photographed, and the knee joints and tibias were observed and photographed after removing soft tissue. (C to E) Then, the tissue was fixed and scanned by micro-CT (C), and the values for porosity (D) and bone mineral density (E) of the tibias were calculated. (F and G) During the treatment, the body weight and temperature of the mice were measured every 2 days. D1 to D7, day 1 to day 7.

Encouraged by the *in vitro* results, the *in vivo* effect of EM+M-LFLIU on osteomyelitis caused by MRSA was also investigated. Each group of mice except the blank group was infected with 10^8^ CFU/mL MRSA. The treatment began on the second day after infection, and the right leg and bone tissue were removed a week later for follow-up experiments (Fig. 6A and B). In contrast to those of the blank group, the right legs of nontreated and M-LFLIU groups were obviously enlarged, with a large number of small pustules scattered on the surface of the muscle tissue visible to the naked eye. The EM group had slightly less swelling in the right leg, as well as a smaller amount of suppurative tissue than the nontreated and M-LFLIU groups. In the EM+M-LFLIU group, the swelling of the right leg was not obvious, and while suppurative tissue could be seen in the knee joint, there were no scattered pustules.

The appearance of bone tissue in various groups was studied after the muscle and soft tissue were removed. The control group, M-LFLIU group, and EM group had more severe bone loss of the knee joint than the blank group, and the tibia appeared enlarged, with suppurative tissue visible in the bone marrow cavity. In the EM+M-LFLIU group, bone destruction was less pronounced, the volume of the tibia was not significantly increased, and the suppurative tissue in the bone marrow cavity was not obvious. The micro-computed tomography (micro-CT) images obtained supported these findings (Fig. 6C). In order to draw a more objective and specific conclusion, we analyzed the bone volume/total volume (BV/TV) ratio and total porosity of bone tissue. The results showed that the BV/TV was lowest in the control group, that it increased after ultrasound or drug treatment, and that the BV/TV in the EM+M-LFLIU group was the closest to that in the blank group, while the total porosity showed the opposite trends, consistent with the micro-CT images (Fig. 6D and E).

In addition, we measured the body temperature and body weight of each group every other day throughout therapy to determine the vital condition of the mice. Contrary to the body weight gain of mice in the blank group, the body weight of untreated mice continuously decreased. The mice treated with M-LFLIU or EM stopped losing weight on the 5th day after treatment, while the mice in the EM+M-LFLIU group had very little weight loss in the early stage. The body weight in the EM+M-LFLIU group started to increase steadily on the 3rd day after treatment and even approached that of the blank group at the end of the treatment period. In terms of body temperature, the temperature of the mice in the nontreated and the M-LFLIU groups reached the highest levels on the 3rd day after treatment and then decreased to approximately 38°C. The average body temperature of the untreated group was higher than that of other groups in all periods. The body temperatures of the mice in the EM and EM+M-LFLIU groups reached above 38°C on the first day of treatment and then gradually decreased to 37.9°C and 37.4°C, respectively. In particular, there was no obvious difference between the EM+M-LFLIU and the blank group on the 7th day after treatment (Fig. 6F and G).

### Antibacterial and anti-inflammatory effects of EM+M-LFLIU *in vivo*.

**FIG 7:**
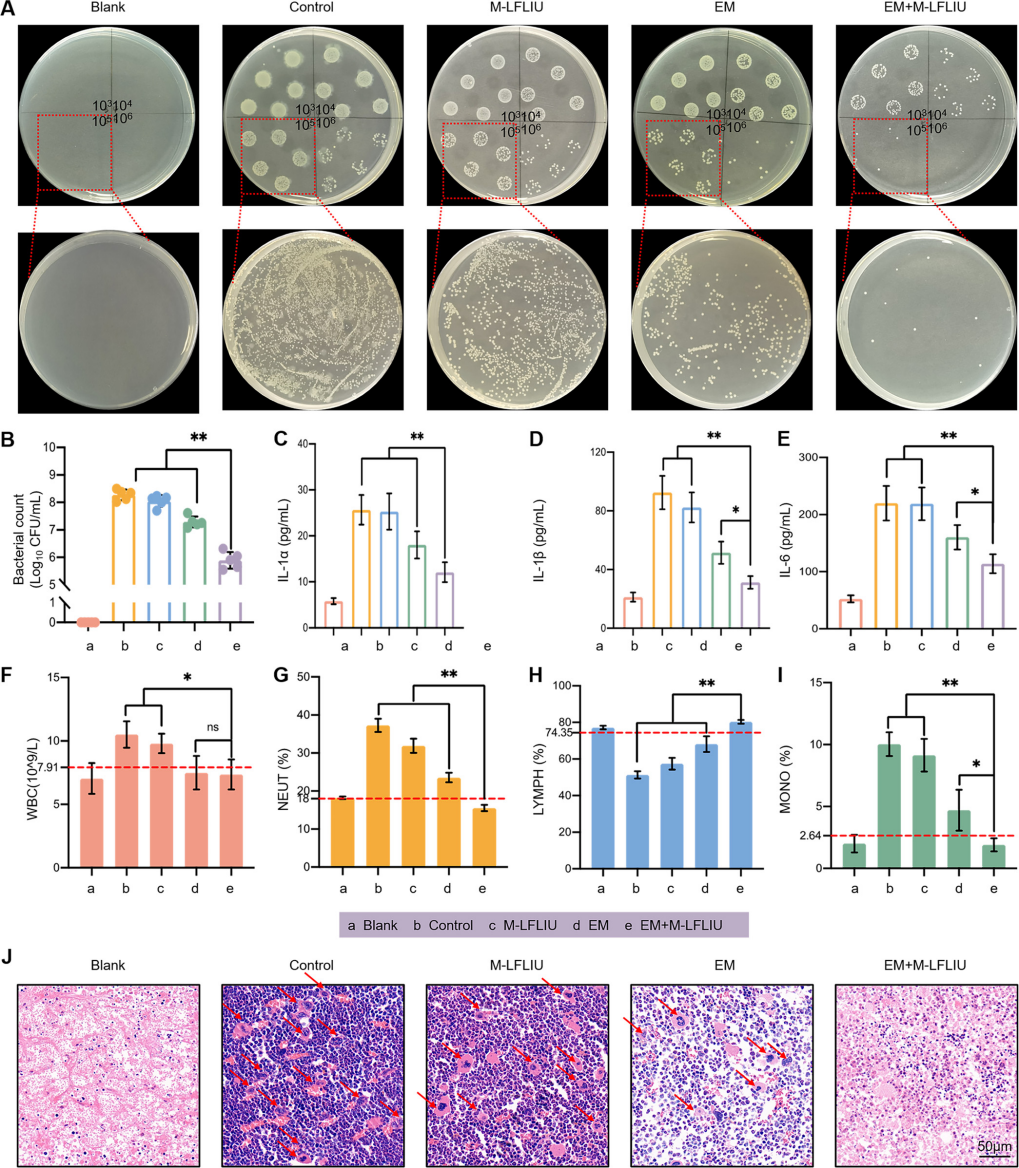
Effects of different treatments on anti-inflammatory germicidal efficacy and hemogram in mice with acute osteomyelitis. (A and B) The tibia tissues of different groups of mice were homogenized, and then the homogenates were used for bacterial spot plates and spread plates to observe the contents of living bacteria in the tissues. (C to E) The remaining tissue homogenates were centrifuged (12,000 rpm for 15 min at 4°C), and the supernatants were used for ELISA detection of different inflammatory factors. (F to J) The blood of each group of mice was taken for blood tests (F to I) and bone tissue hinge staining (J), and the changes of inflammatory cells in different groups were observed. WBC, white blood cells; NEUT, neutrophils; MONO, monocytes.

To further quantify the effects of each treatment on the different groups of mice, the bones of the previously collected right legs of the mice were ground and homogenized at high speed, and then the homogenate was diluted. The effects of the treatments were evaluated via bacterial plate counting and enzyme-linked immunosorbent assay (ELISA). Pictures of colonies on the LB agar plates and the quantitative statistics of the colony count numbers are shown in Fig. 7A and B. The ELISA results for inflammatory factors in bone tissue are shown in Fig. 7C to E.

In comparison to the untreated group, the number of colonies and the expression levels of interleukin-1α (IL-1α), IL-1β, and IL-6 were slightly lower in the M-LFLIU group and evidently reduced in the EM group and EM+M-LFLIU groups. Moreover, the EM+M-LFLIU group had the smallest number of colonies and the lowest levels of inflammatory factors. Consistent with these findings, hematoxylin and eosin (H&E) staining of bone tissue sections also showed that the control group had a large number of inflammatory cells, suggesting severe inflammation, and the inflammation in the EM+M-LFLIU group was greatly alleviated (Fig. 7J).

Finally, a complete blood count was performed to estimate the degree of inflammation in the mice. The blood test results indicated that the inflammation of the EM group was suppressed to a certain extent, while the inflammation of the EM+M-LFLIU group mice was effectively controlled (Fig. 7F to I). As the degree of inflammation in mice intensifies, the number of leukocytes and the ratio of neutrophils and monocytes will increase significantly, while the ratio of lymphocytes will decrease. Therefore, we suggest that EM+M-LFLIU has excellent potential for effectively killing bacteria and decreasing the levels of inflammatory markers.

### Pilot toxicity testing *in vivo*.

**FIG 8:**
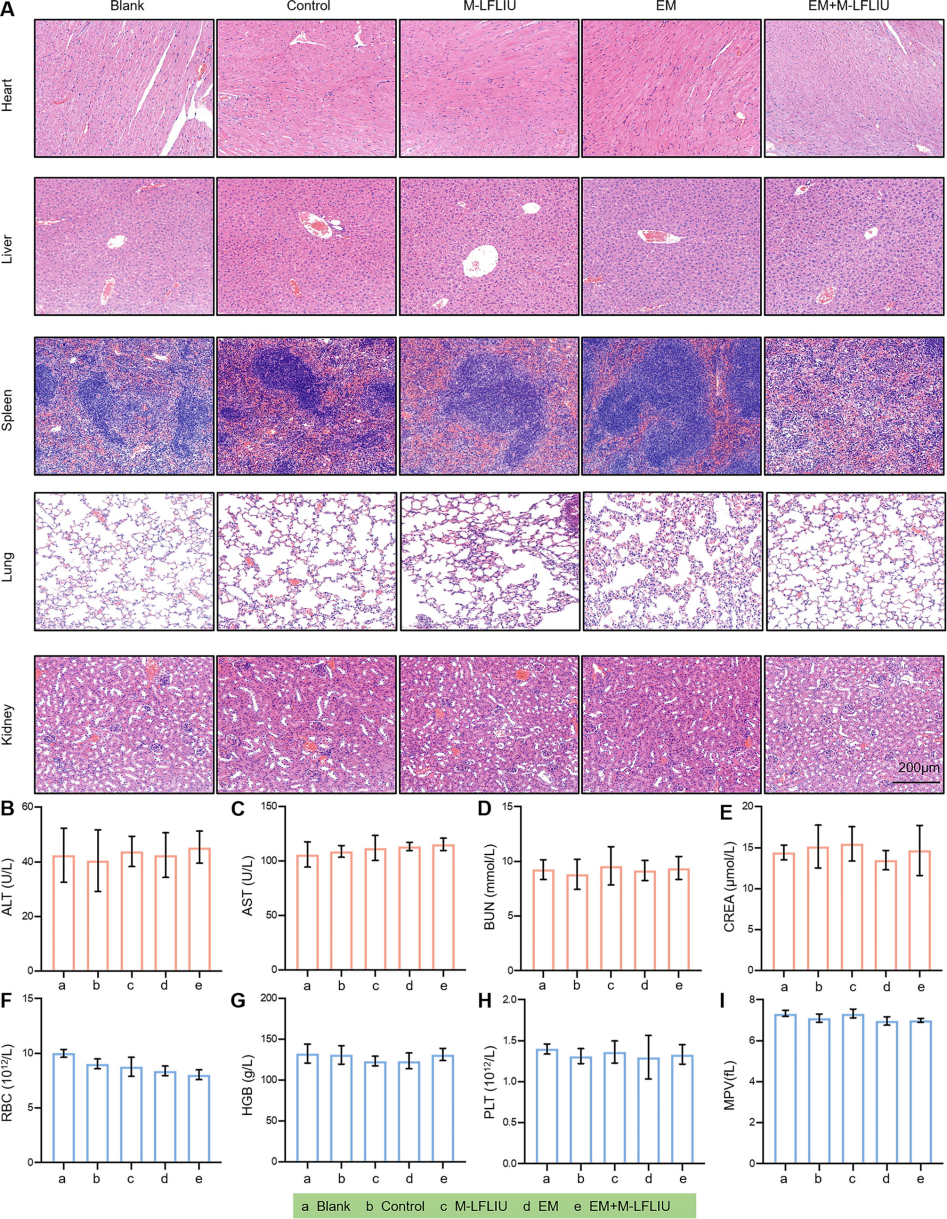
Pilot toxicity testing *in vivo*. (A) Two weeks after the treatment, the heart, liver, spleen, lung, and kidneys were stained with H&E for the determination of toxicological histology. (B to I) Blood biochemistry markers alanine aminotransferase (ALT) (B), aspartate aminotransferase (AST) (C), blood urea nitrogen (BUN) (D), and creatinine (CREA) (E) and complete blood count, including red blood cells (RBC) (F), hemoglobin (HGB) (G), platelets (PLT) (H), and mean platelet volume (MPV) (I), were tested to explore the toxicity of EM and M-LFLIU *in vivo*.

We then tested whether treatment with EM plus M-LFLIU had a toxic effect on mice. The histological structures of the heart, liver, spleen, lung, and kidney (Fig. 8A), the complete blood count, and the hepatic/renal function indices (Fig. 8B to I) all indicated that EM and M-LFLIU treatment did not induce any significant alterations in mice. These results suggest that EM+M-LFLIU has excellent biosafety at the therapeutically efficacious dose *in vivo*.

## DISCUSSION

Traumatic osteomyelitis, which often develops a MSSA infection, is the most common type of osteomyelitis (34). Vancomycin is recognized as an effective drug for the clinical treatment of acute osteomyelitis (35). However, the formation of bacterial biofilms can reduce the effectiveness of antibiotics. With the increasing prevalence of antibiotic resistance in bacteria (36), developing new antibacterial drugs and alternative therapies is imperative. EM is a drug with significant anti-inflammatory and antibacterial activity (37, 38), and thus, it has the potential to replace antibiotics as an effective drug for treating acute osteomyelitis. Previous studies have reported the antibacterial effect of ultrasound in food packaging (39), and it has also been reported to stimulate ROS production, which provides a specific repressive effect on the growth of suspended bacteria and bacterial biofilms (12, 13, 40). In this study, it was found that M-LFLIU combined with EM had significant antibacterial effects both *in vitro* and *in vivo*, providing a promising possibility for alternative clinical treatments of acute osteomyelitis.

The formation of bacterial biofilm is widely regarded as an important part of the bacterial infection process and a major challenge in treating an infection (41). From the above-described results, it is seen that our experiments validate and extend the previous hypothesis that LFLIU and EM would have combined antibacterial activity. In this verification, we found that the combination of LFLIU and EM, especially M-LFLIU and EM, significantly inhibited the growth of bacteria and the formation of bacterial biofilm and increased the destruction of biofilm structure under conditions of constant ultrasonic intensity and EM concentration. This effectively weakens the barrier effect of the bacterial biofilm so that the drug can pass through the bacterial barrier at a low concentration and, thus, reduces the toxicity and side effects caused by high concentrations of drugs.

In order to explore the related antibacterial mechanism, we used transmission electron microscopy to observe whether EM combined with M-LFLIU could effectively lead to the rupture of the bacterial cell wall. Because human cells have no cell wall structure, this antibacterial therapy does less damage to human cells. Because EM has broad-spectrum effects, including anti-inflammatory, antioxidant, and antibacterial effects (42), we used a confocal probe and flow cytometry to detect whether M-LFLIU could enhance the antibacterial effect of EM-stimulated ROS. In the process of the body’s fight against bacteria, proper ROS promotes the body’s immunity and repair. The research on therapeutic use of EM combined with M-LFLIU shows that it can enhance the body’s immune response to bacteria to a certain extent.

In addition, the PCR results suggest that EM+M-LFLIU can reduce the virulence of MRSA, reduce its toxic side effects on the host, weaken the resistance of MRSA to oxidative stress, and enhance the germicidal efficacy of ROS. Thus, it can be seen that EM combined with M-LFLIU not only has effective germicidal efficacy but also has a significant weakening effect on the surviving bacteria, which can improve the condition of patients with osteomyelitis in a short time. At the same time, these experimental results suggest the potential antibacterial mechanism of EM, which provides a direction for the study of the mechanism of LFLIU combined with EM in the clinical treatment of bacterial infection.

In the animal experiments, compared with the traditional methods of intragastric administration or intraperitoneal injection, emodin aseptic saline was used to rinse the wound and make a wet compress that was applied locally, which effectively increased the local effective drug concentration, avoided the possible toxic and side effects caused by a systemic overdose of emodin, combined better with ultrasound to form local targeting, and was closer to the clinical treatment of osteomyelitis. It has higher efficiency and better prospects in scientific research and clinical conversion. What is more exciting is that the symptoms of osteomyelitis in mice treated with M-LFLIU combined with EM were significantly alleviated, the degree of bone destruction was mild, inflammatory indicators and vital signs recovered quickly, and there were no obvious side effects. This seems to be the expected ideal result.

For future research, we put forward several research directions, as follows: (i) further exploration of the antibacterial molecular targets and molecular mechanisms of EM combined with M-LFLIU, especially the genes and targets of oxidative stress; (ii) exploration of the antibacterial effect of EM combined with M-LFLIU on other bacteria and the antibacterial effect of other sound sensitizers combined with LFLIU on MRSA and of the mechanism behind the phenomenon; and (iii) on the basis of this study, exploration of the addition of new physiotherapies, such as sound sensitizers combined with nano-copper beads that could generate heat and copper ions to kill bacteria under the action of ultrasound, which may further improve the therapeutic effect. Thus, it can be seen that the current research has made a good start and there is a broad space for follow-up research.

In a word, the antibacterial effect of M-LFLIU combined with EM promises to be an effective and promising clinical treatment. It is expected to solve the problems of the difficulty in using antibiotics to solve the problem of biofilm disturbance and the production of drug-resistant bacteria.

Unfortunately, our results at this stage are still obviously incomplete. First, the number of genes screened by PCR was limited, and we only tentatively looked for several classic pathogenic genes of MRSA for PCR amplification, which likely missed expressed genes that were really affected. Second, we did not detect whether the protein content associated with other PCR-negative genes was changed, and we could not detect whether the RNA translation and protein synthesis were affected. However, due to the limitations of funds, time, researchers’ ability, and other objective factors, the follow-up experiments have not been carried out accordingly. It may be a good plan to increase the ELISA detection of related proteins in the follow-up study.

In summary, our study expands the body of knowledge on the antibacterial effect of drugs—specifically of EM—through combined physiotherapy. If successfully integrated into clinical practice, these methods may reduce the burden of high concentrations of drugs needed to treat bacterial biofilms and avoid the growing resistance of bacteria to antibiotics. We have shown that this method provides a meaningful possibility for treating osteomyelitis caused by S. aureus, especially osteomyelitis caused by MRSA. Future research may show that it can be useful in treating infections and conditions caused by other antibiotic-resistant bacteria.

## MATERIALS AND METHODS

### Strains, reagents, and instruments.

Methicillin-sensitive Staphylococcus aureus (MSSA; strain ATCC 29213) and methicillin-resistant Staphylococcus aureus (MRSA; strain ATCC 43300) were obtained from the American Type Culture Collection. LB broth and LB agar were purchased from Sangon Biotech (Shanghai, China). Distilled deionized water was obtained from a Millipore purification system (MilliporeSigma, USA). EM was acquired from Meilun Biotech (Dalian, China). Crystal violet was bought from Beyotime (Shanghai, China). The LIVE/DEAD bacterial viability kit was procured from Invitrogen Molecular Probes (Eugene, OR, USA).

The LFLIU instrument (Nu-Tek Soni-Stim pro UT1021), with adjustable dual frequencies (1 MHz and 3 MHz), effective radiation area of 5.0 cm^2^, and adjustable output power of 0.1 to 3 W/cm^2^, was purchased from Kang Zan Medical Device Technology Co., Ltd. The OD values were measured using an M5 multifunctional microplate reader (SpectraMax M5; Molecular Devices, USA), fluorescence photos were taken using an SP8 Lightning confocal microscope (Leica, Germany), and the microstructure of bacteria was photographed using a transmission electron microscope (TEM) (Hitachi H-7650).

### *In vitro* assessment of antimicrobial activity.

MSSA and MRSA were grown in LB broth medium at 37°C for 12 h. The bacterial suspensions were then diluted to 10^6^ CFU/mL for the succeeding experiments.

For the ultrasonic sterilization experiment, 1-mL amounts of the bacterial dilutions were added into the 24-well plates. S-LFLIU refers to continuous irradiation for 15 min at 1 MHz frequency, and intensities of 0.1, 0.3, 0.5, and 1 W/cm^2^ were tested. The operational frequency and intensity of M-LFLIU were the same, but the setup was irradiated for 5 min every 4 h (three times a day [TID]). After corresponding irradiation, the treated bacteria were incubated at 37°C for 6 h or 24 h to obtain the OD_600_ value to calculate the survival rate of the bacteria. After these initial experiments, the ultrasonic intensity of 1 W/cm^2^ was found to induce the best antibacterial effect and was used in all succeeding experiments.

To determine the optimal concentration of EM, 50 μL double-diluted EM (final concentrations of 1.56, 3.13, 6.25, and 12.5 μg/mL) and 50 μL bacterial solution were added to 96-well plates and incubated at 37°C for 24 h before OD_600_ measurement. For the combined antibacterial experiments, 500 μL bacterial suspension and 500 μL double-diluted EM (final concentrations of 1.56, 3.13, 6.25, and 12.5 μg/mL) were added into 24-well plates and then treated with S-LFLIU (1 W/cm^2^ for 15 min) and M-LFLIU (1 W/cm^2^ for 5 min TID). Then, the treated bacteria were incubated (37°C for 24 h) and the OD_600_ was measured.

We then divided the samples into six treatment groups for quantitative analysis: the control group, the S-LFLIU group, the M-LFLIU group, the EM group, the EM+S-LFLIU group, and the EM+M-LFLIU group (EM, 6.25 μg/mL; S-LFLIU, 1 W/cm^2^ for 15 min; and M-LFLIU, 1 W/cm^2^ for 5 min TID). At 24 h following treatment, the bacterial suspensions were collected, diluted serially from 10^1^ to 10^4^ times, and point inoculated onto LB agar plates (10 μL per point and 5 points for each concentration). In addition, 100-μL amounts of the 10^3^ dilutions were placed onto separate LB agar plates and spread evenly. These LB agar plates were incubated (37°C for 24 h) for colony counting.

### Live/dead staining assay.

MRSA suspensions (10^8^ CFU/mL) were treated with blank LB medium, S-LFLIU alone, M-LFLIU alone, EM alone, EM+S-LFLIU, or EM+M-LFLIU (EM, 6.25 μg/mL; S-LFLIU, 1 W/cm^2^ for 15 min; and M-LFLIU, 1 W/cm^2^ for 5 min TID). After 3 h of incubation at 37°C, bacterial suspensions were centrifuged (5,000 rpm for 10 min at 4°C), resuspended in SYTO 9/propidium iodide (PI) (green/red) dye, and then incubated at 37°C for 30 min. After that, 10-μL amounts of the bacterial suspensions were dropped onto slides to observe the fluorescence signals under an SP8 Lightning confocal microscope (Leica, Germany).

### TEM analysis.

MRSA suspensions (10^8^ CFU/mL) were treated with blank LB medium, S-LFLIU alone, M-LFLIU alone, EM alone, EM+S-LFLIU, or EM+M-LFLIU (EM, 6.25 μg/mL; S-LFLIU, 1 W/cm^2^ for 15 min; and M-LFLIU, 1 W/cm^2^ for 5 min TID). After 12 h of incubation at 37°C, the bacterial suspensions were centrifuged (5,000 rpm for 10 min at 4°C) and fixed overnight at 4°C in 2.5% glutaraldehyde solution. They were then washed three times in phosphate-buffered saline solution (0.1 M, pH 7.0) (PBS), followed by a series of fixation, dehydration, permeation, embedding, sectioning, and staining steps. Finally, the bacteria were observed under a TEM.

### Biofilm formation and antibiofilm activity.

To determine whether the EM with LFLIU could destroy formed biofilms, the suspensions of MRSA were diluted to 10^7^ CFU/mL and cultivated in 24-well plates at 37°C for 24 h to allow the formation of biofilms. After this, the culture supernatants were discarded, and the mature biofilms were treated with S-LFLIU alone, M-LFLIU alone, EM alone, EM+S-LFLIU, or EM+M-LFLIU (EM, 6.25 μg/mL; S-LFLIU, 1 W/cm^2^ for 15 min; and M-LFLIU, 1 W/cm^2^ for 5 min TID). After 24 h of treatment at 37°C, the bacterial solution was removed and the remaining biofilm was washed three times in PBS solution to remove planktonic bacteria. Afterward, the residual biofilms were stained with crystal violet (200 μL/well) for 20 min and then washed in PBS solution three times to remove uncombined crystal violet. Air-dried biofilms were photographed, and then combinative crystal violet dye was dissolved by adding 1 mL 95% ethanol to each well and the biofilm was quantified by measuring the OD_570_ of the solution. Finally, the damage rate in the biofilms was calculated by comparing the OD_570_ values of the dissolved biofilms using the following formula: % destruction = [(OD_570_ control − OD_570_ sample)/OD_570_ control] × 100.

After a similar procedure of treatment, incubation, and washing, the formed biofilms were collected, dispersed via ultrasonication (ultrasonic cleaning machine at 30 kHz) for 30 min, and then diluted serially from 10^2^ to 10^5^ times. These dilutions were point inoculated onto LB agar plates (10 μL per point and 5 points for each concentration), and meanwhile, 100-μL amounts of the 10^4^ dilutions were point inoculated onto separate LB agar plates and spread evenly. These LB agar plates were incubated at 37°C for 24 h for colony counting.

Cover glasses were placed at the bottom of the wells of 6-well plates, and 2 mL of suspended MRSA solution (10^7^ CFU/mL) was introduced into each well and incubated for 24 h at 37°C to form biofilms on the glass slides. After a similar procedure of treatment, incubation, and washing, the biofilm was stained with SYTO 9 dye for 30 min and its 3-D structure was observed under a confocal microscope.

To test the inhibition and eradication effects, EM and LFLIU were applied against MRSA suspensions (10^7^ CFU/mL) at 0 h and biofilms at 24 h. Then, the residual biofilms were evaluated via crystal violet staining, plate counting, and SYTO 9 staining similarly to the aforementioned methods.

### ROS detection.

MRSA suspensions (10^8^ CFU/mL) were added into 6-well plates and treated with blank LB, EM, M-LFLIU, or EM+M-LFLIU (EM, 6.25 μg/mL, and M-LFLIU, 1 W/cm^2^). After treatment, the suspensions were incubated at 37°C for 3 h, and the bacterial cells were collected, stained with DCFH-DA (2′,7′-dichlorodihydrofluorescein diacetate; Yeasen, Shanghai, China) at 37°C for 30 min, and imaged under a confocal microscope. The ROS-positive rates for each sample were measured by a CytoFlex S flow cytometer (Beckman Coulter, USA).

### qRT-PCR.

The primers used in our study are listed in Table 1. After treatment with EM, M-LFLIU, or EM+M-LFLIU (EM, 6.25 μg/mL, and M-LFLIU, 1 W/cm^2^), MRSA bacteria were collected, resuspended in a solution containing 3 mg/mL lysozyme diluted in Tris-EDTA [TE] buffer (Meilun Biotech), and incubated at room temperature for 10 min for bacterial lysis. The RNApure bacteria kit (Conway Biotech, Beijing) was then used to extract the mRNA of the bacteria following the manufacturer’s directions, and the mRNA was then reverse transcribed into cDNA (TaKaRa, Beijing). Quantitative real-time PCR (qRT-PCR) was performed using a CFX96 PCR detection system (Bio-Rad, USA) and a SYBR green I mixture (TaKaRa, Beijing). The MRSA housekeeping gene *gyrB* was used as an internal reference for normalization of the data (Table 1).

**TABLE 1:** Sequences of primers for qRT-PCR

Gene	Primer direction	Primer (5′–3′)
*agrB*	Forward	TATGCAAGCCAAGCACTTGT
	Reverse	GTGCGAAAGCCGATAACAAT
*pbp3*	Forward	TCAAATGATGAAATCGTTCAAAA
	Reverse	TCCGATTGTGTTGTTTTTCG
*sgtB*	Forward	GCGATAATGTGGATGAACTAAG
	Reverse	TTGGTCCTTAGCCACTCCTTC
*gmk*	Forward	TCGTTTTATCAGGACCATCTGGAGTAGGTA
	Reverse	CATCTTTAATTAAAGCTTCAAACGCATCCC
*zwf*	Forward	CGCGTTGCAGATGACTCTAA
	Reverse	AATGGTACACCAGCCCATCT
*msrA*	Forward	GGCACAATAAGAGTGTTTAAAGG
	Reverse	AAGTTATATCATGAATAGATTGTCCTGTT

### Animal experiments.

In this study, 12-week-old male C57BL/6 mice (weighing 20 to 25 g) obtained from Shanghai SLAC Laboratory Animal Co., Ltd., were used to study the effect of M-LFLIU combined with EM on MRSA-induced osteomyelitis. Animal care and experiments were carried out in accordance with the guidelines and procedures authorized by the Animal Experimental Ethics Committee of the Taizhou Hospital of Zhejiang University. After adapting to the experimental conditions (control diet) for 1 week, the mice were treated with a sham operation (blank group; *n* = 6) or an osteomyelitis model (experimental group; *n* = 24).

An overnight culture of MRSA was prepared in LB and adjusted to 10^8^ CFU/mL. After centrifugation (5,000 rpm for 10 min at 4°C), the pellet was resuspended in PBS solution. Before the operation, the mice were anesthetized with isoflurane. The right knee joint was shaved and sterilized, and a small incision was made on the outside of the knee joint. The tibial plateau was exposed via blunt anatomy, and a single cortical bone defect 1.5 mm in diameter was made using a dental drill. After inoculating 10 μL of bacteria into the marrow of the right bone defect, the muscle fascia and skin were sutured. All mice were maintained on a standard diet and had free access to sterile water. After the operation, which was designated day 0, the animals were divided equally into the following groups: (i) the blank group, treated with LB broth; (ii) the control group, with no treatment; (iii) the ultrasound treatment group, treated with M-LFLIU therapy (1 W/cm^2^ for 5 min TID); (iv) the drug treatment group, treated with EM; and (v) the combined treatment group, treated with M-LFLIU (1 W/cm^2^ for 5 min TID) and EM. The treatment was performed according to the following procedure. Sutures were removed from the surgical incision of the mouse to expose the tibial bone hole. Ten milliliters of EM solution (containing 400 μg EM) was used to continuously rinse the wound and bone lesion (fluid flow rate, 10 mL/min) for about 1 min, during which ultrasound (1 W/cm^2^ for 5 min TID) was used on the wound. The wound was then wet with gauze soaked in EM solution and bandaged with aseptic dressing. During this period, the body temperature and body weight of mice were measured every other day. After 7 days, the right legs and bone samples of mice were collected for further experiments.

### Evaluation of the effect of EM and M-LFLIU on osteomyelitis *in vivo*.

For the quantitative analysis of bacteria in experimental animal tissue, the right legs of the mice were removed 7 days after the operation, as mentioned above. The muscle and soft tissue were subsequently removed, and only the bone tissue was retained. After adding 1.5 mL PBS solution, a homogenate was obtained via high-speed oscillatory grinding. This homogenate was serially diluted and point inoculated onto LB agar plates (10 μL per point and 5 points for each concentration). The tissue homogenate was then diluted 10^5^ times, and 100 μL was placed on an LB agar plate and spread evenly. These LB agar plates were incubated at 37°C for 24 h to count the number of colonies.

### Micro-CT analysis.

The tibias and knee bones of mice were scanned with high-resolution micro-computed tomography (micro-CT) (μCT-100; Scanco Medical Co., Ltd.) at 20-μm resolution, 70 kV, and 200 μA, with an exposure time of 300 ms. After three-dimensional reconstruction of bone tissue, the degrees of damage to the above-mentioned structures in the mice in the different groups were observed and compared. The total porosity and BV/TV (bone volume/total volume) of each sample were evaluated by using Scanco software version 6.5-3 (Scanco Medical AG, Switzerland).

### ELISA.

The right lower leg bones of mice were pulverized and homogenized at high speeds, and the homogenate was used in ELISA studies. ELISA kits provided by Ameko were used to evaluate the expression levels of IL-6, IL-1, and IL-1 in the homogenate (Hangzhou, China). All the procedures were performed in compliance with the manufacturer’s instructions.

### *In vivo* toxicity assay.

Two weeks after the treatment, routine blood and biochemical tests were conducted to explore the toxicity of EM and M-LFLIU *in vivo*. The heart, liver, spleen, lung, and kidneys were fixed with 4% formaldehyde, dehydrated, embedded in paraffin, and sliced to a thickness of 5 μm. The sections were then stained with H&E for the determination of toxicological histology.

### Statistical analysis.

The results from three or more independent experiments were expressed as the average value ± standard deviation (SD). For intergroup comparison, we used Student’s *t* test (unpaired, two tailed). The value was expressed as the mean ± standard error of the mean (SEM), and a *P* value of <0.05 was considered statistically significant (*, *P* < 0.05, and **, *P* < 0.01).

### Ethics statement.

The study was conducted according to the guidelines of the Declaration of Helsinki and approved by the Institutional Animal Ethics Committee of Taizhou Hospital (protocol code tzy-2021164).

### Data availability.

The raw data supporting the conclusions of this article will be made available by the authors, without undue reservation.
