# Supplementary material for: Emodin Combined with Multiple-Low-Frequency, Low-Intensity Ultrasound To Relieve Osteomyelitis through Sonoantimicrobial Chemotherapy
Source: Microbiol Spectr. 2022 Sep 7;10(5):e00544-22. doi: 10.1128/spectrum.00544-22 (PMC9603654; doi:10.1128/spectrum.00544-22)
Supplement: Supplemental file 1 — Download spectrum.00544-22-s0001.pdf, PDF file, 0.5 MB [file spectrum.00544-22-s0001.pdf]

Supporting Information

Emodin Combined with Multiple Low-frequency Low-intensity Ultrasound to Relieve Osteomyelitis through Sonoantimicrobial Chemotherapy

Feng Lu[1,2] †, Xinhui Wu[3,4] †, Huiqun Hu[2,5], Zixuan He[1,2], Jiacheng Sun[3,4], Jiapeng Zhang[3,4], Xiaoting Song[3,4], Xiangang Jin[1,2] and Guofu Chen[4,*]

[1] Department of Orthopedic, Taizhou Hospital of Zhejiang Province, Zhejiang University, Linhai, 317000, China

[2] Zhejiang University School of Medicine, Hangzhou, 310009, China

[3] Wenzhou Medical University, Wenzhou, 325035, China

[4] Department of Orthopedic, Taizhou Hospital Affiliated to Wenzhou Medical University, Linhai, 317000, China

[5] Department of Infectious Diseases, The Second Affiliated Hospital, Zhejiang University School of Medicine, Hangzhou, 310009, China

[†] **These authors contributed equally.**

***Correspondence:**
Guofu Chen
chenguofu@enzemed.com

Supporting Methods

Antibacterial Resistance Test

Firstly, the MIC of EM+M-LFLIU and ampicillin (AMP) against MRSA (generation 0) was tested to evaluate the MIC before antibacterial treatment. Then, the MRSA were treated with EM +M-LFLIU or AMP at a concentration of half MIC, and the surviving bacteria were identified as the 1st-generation cells. MIC of the 1st-generation cells was then tested, and then the MRSA that survived after half of MIC (1st generation) treatment were identified as the 2nd-generation cells. These operations were repeated for 6 generations, and the MIC changes of each generation were used to evaluate the effect of EM+M-LFLIU or AMP on the drug resistance of MRSA.

Supporting Figures

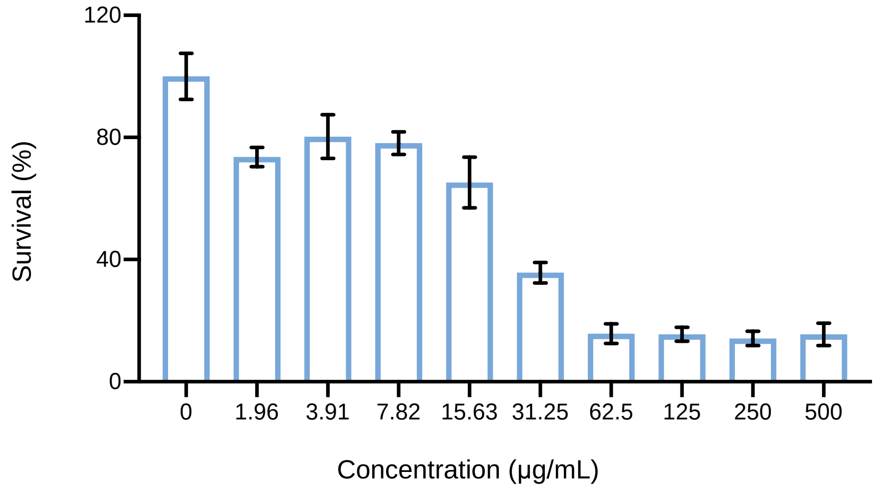

FIGURE S1. The survival rate of and MRSA bacterial cells after AMP treatment.

The survival rate of MRSA treated with different concentrations of AMP after 24 h.

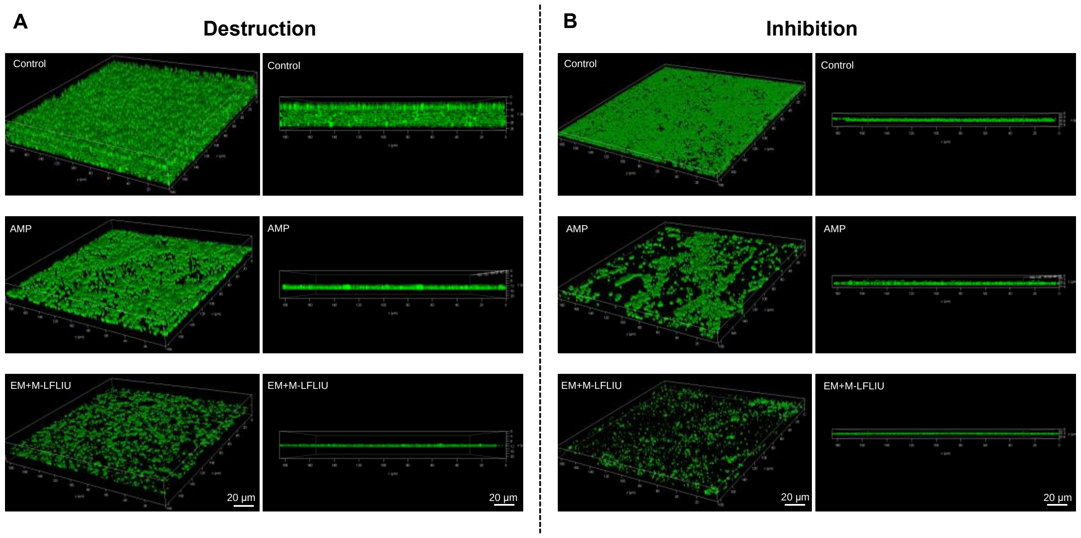

FIGURE S2. The 3D structures of immature and mature biofilms treated with AMP and EM+M-LFLIU.

Biofilms were formed on the glasses, treated with corresponding treatment, stained with SYTO 9 to obtain 3D fluorescence image of mature MRSA biofilm **(A)** and immature MRSA biofilm (left: top view right: side view) **(B)**.

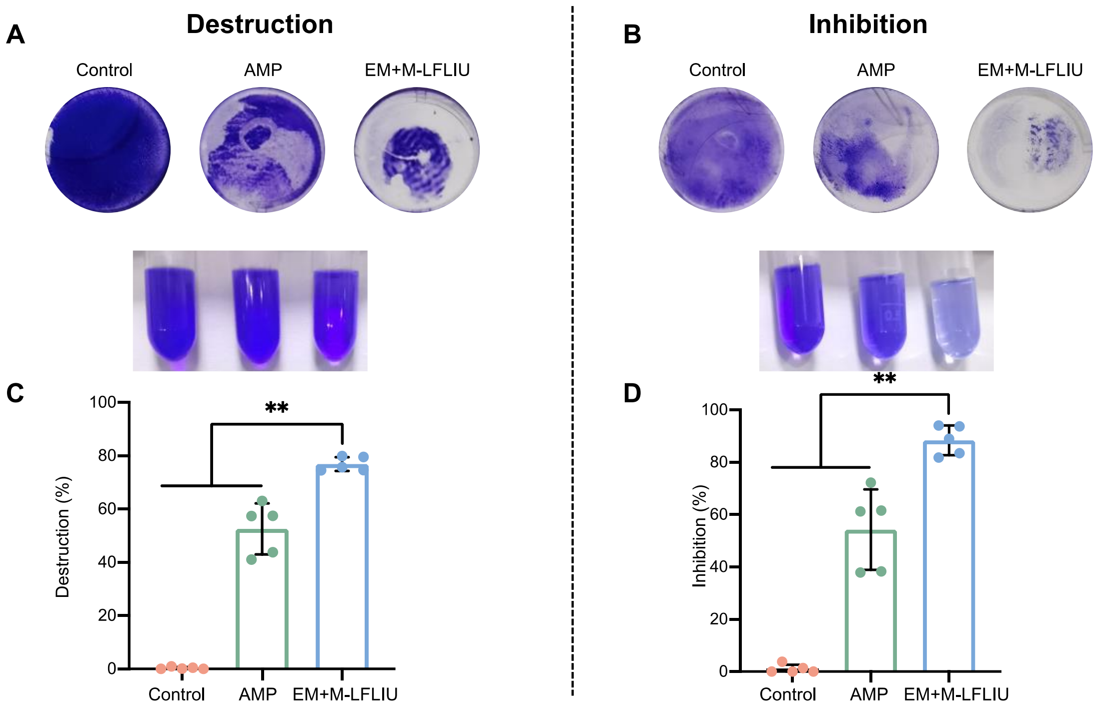

FIGURE S3. The The 3D structures of immature and mature biofilms treated with AMP and EM+M-LFLIU. of immature and mature biofilms treated with AMP and EM+M-LFLIU.

MRSA were treated with AMP or EM+M-LFLIU respectively at 37 °C for 24 h (M-LFLIU: 5 min every 4 h, three times), then bacteria solution was discarded, residual biofilms were stained by crystal violet staining **(A)**. MRSA was incubated at 37 °C for 24 h to form mature biofilm, then mature biofilms treated with AMP or EM+M-LFLIU respectively at 37 °C for 24 h (M-LFLIU: 5 min every 4 h, three times), and then stained with crystal violet **(B)**. The destruction rate **(C)** and inhibition rate **(D)** of MRSA biofilm formation was calculated (upper: photo of crystal violet-stained biofilm dissolved in ethanol)

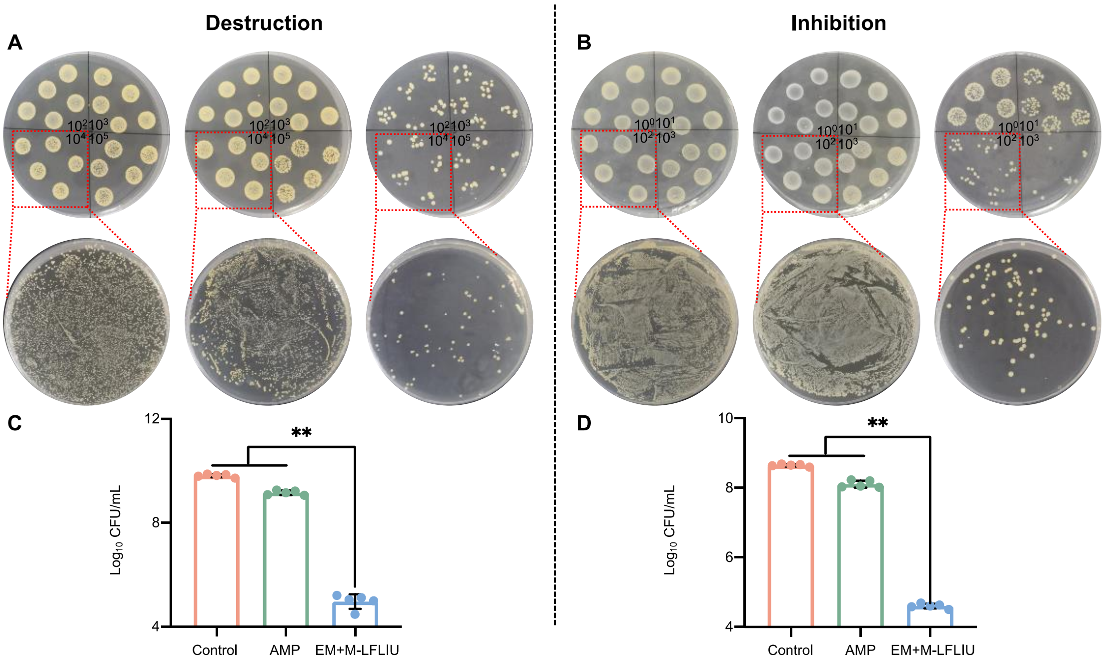

FIGURE S4. Living bacteria activity in biofilm after treated with AMP or EM+M-LFLIU.

MRSA was incubated at 37 °C for 24 h to form mature biofilm, then mature biofilms treated with AMP or EM+M-LFLIU respectively at 37 °C for 24 h (M-LFLIU: 5 min every 4 h, three times), the living MRSA grew on the LB agar plates (top: diluted 10^2, 10^3, 10^4, 10^5 times, bottom: diluted 10^4 times) **(A)**. MRSA were treated with AMP or EM+M-LFLIU respectively at 37 °C for 24 h (M-LFLIU: 5 min every 4 h, three times), residual biofilms were collected, dispersed, grew on the LB agar plates (top: diluted 10^0, 10^1, 10^2, 10^3 times, bottom: diluted 10^2 times) **(B)**. The number of living bacteria in both mature **(C)** and immature **(D)** biofilm were qualified.

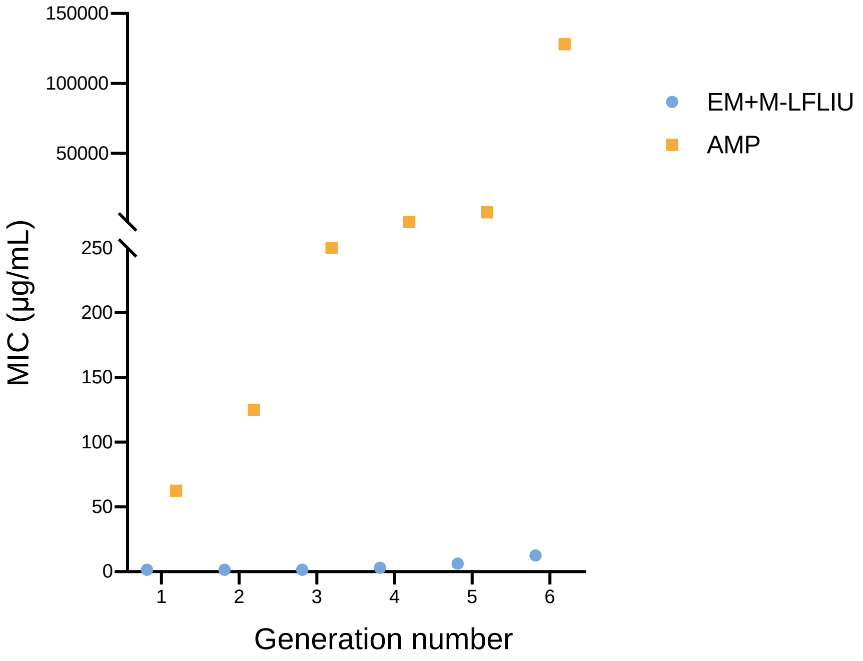

FIGURE S5. Resistance of MRSA to AMP and EM+M-LFLIU.

Resistance development of MRSA after continuous exposure toward AMP or EM+M-LFLIU at the concentration of half MIC for 6 passages.
